# The tyrosine kinase Yes1 is a druggable host factor of HEV

**DOI:** 10.1097/HC9.0000000000000553

**Published:** 2024-10-17

**Authors:** Jil Alexandra Haase, Abarna Baheerathan, Xin Zhang, Rebecca Menhua Fu, Maximilian Klaus Nocke, Charlotte Decker, Viet Loan Dao Thi, Daniel Todt, Johan Neyts, Suzanne J.F. Kaptein, Eike Steinmann, Volker Kinast

**Affiliations:** 1 Department of Molecular and Medical Virology, Faculty of Medicine, Ruhr University Bochum, Bochum, Germany; 2 Institute of Clinical and Molecular Virology, University Hospital Erlangen, Friedrich-Alexander-University Erlangen-Nürnberg, Erlangen, Germany; 3 KU Leuven Department of Microbiology, Immunology and Transplantation, Rega Institute, Laboratory of Virology and Chemotherapy, Leuven, Belgium; 4 Schaller Research Group, Department of Infectious Diseases and Virology, Heidelberg University Hospital, Heidelberg, Germany; 5 Heidelberg Biosciences International Graduate School, Heidelberg University, Heidelberg, Germany; 6 German Centre for Infection Research (DZIF), Partner Site Heidelberg, Heidelberg, Germany; 7 European Virus Bioinformatics Centre (EVBC), Jena, Germany; 8 German Centre for Infection Research (DZIF), External Partner Site, Bochum, Germany; 9 Department of Medical Microbiology and Virology, Carl von Ossietzky University Oldenburg, Oldenburg, Germany

**Keywords:** antivirals, c-Yes, HEV, host factor, life cycle, viral entry, viral pathogenesis, Yes1

## Abstract

**Background:**

HEV is a positive-sense, single-stranded RNA virus of the *Hepeviridae* family. Although HEV accounts for more than 3 million symptomatic cases of viral hepatitis per year, specific anti-HEV therapy and knowledge about HEV pathogenesis are scarce.

**Methods:**

To gain a deeper understanding of the HEV infectious cycle and guide the development of novel antiviral strategies, we here used an RNAi mini screen targeting a selection of kinases, including mitogen-activated protein kinases, receptor tyrosine kinases, and Src-family kinases. Further, we used state-of-the-art HEV infection models, including primary human hepatocytes and athymic nude rats.

**Results:**

Upon knockdown of the Src-family kinase Yes1, a significant reduction of HEV susceptibility could be observed, suggesting an important role of Yes1 in the HEV infectious cycle. Selective inhibition of Yes1 kinase activity resulted in significant inhibition of HEV infection in hepatoma cells and primary human hepatocytes, as well as in a rat HEV *in vivo* model system. Subsequent analysis of Y1KI during the HEV infectious life cycle indicated a role of Yes1 kinase activity in the early onset of HEV infection.

**Conclusions:**

We identified the dependence of HEV on Yes1 signaling, which may contribute to the so far scarce knowledge of HEV’s pathogenesis in the future. Moreover, we provide Y1KI as a novel antiviral drug candidate specifically targeting an HEV host factor.

## INTRODUCTION

As a leading cause of acute viral hepatitis worldwide, HEV poses a significant health burden. Depending on the genotype, HEV can either cause waterborne outbreaks in areas with lower hygienic standards or spread as a zoonotic infection, especially through the food chain, to humans. This results in an estimated 20 million infections and 44,000–70,000 deaths per year.^1^ Clinical manifestations of HEV infections are diverse, ranging from mild, acute hepatitis symptoms up to fulminant hepatitis and chronic infections in immunocompromised individuals.^2^ Noteworthy, pregnant women are at a high risk for fatal outcomes upon HEV infection, with case fatality rates up to 30%.^2^,^3^ Despite these major problems, our current understanding of HEV pathogenesis remains limited and there is a need for improvement of anti-HEV therapy.

Efficient viral replication depends on various virus-caused alterations and manipulations of the cell, resulting in significant changes in the host cell. In this process, kinases are of particular importance because they can rapidly modulate the activity of signaling molecules by (de-)phosphorylating them, thereby enabling the activation or inactivation of the underlying pathways. Different viruses, including human cytomegalo virus,^4^ HBV,^5^ and HCV^6^,^7^, have been reported to regulate kinases and cellular core signaling pathways to repurpose and manipulate the host cell metabolism. For example, HCV triggers a reduced phosphorylation of AMP-activated protein kinase enabling lipid accumulation in virus-infected cells and consequently perturbs the cholesterol and/or fatty acid biosynthesis.^8^ Apart from ensuring viral replication, these molecular changes may also contribute to more global effects, like the virus-caused pathophysiology highlighted by HCV-triggered steatosis.^9^,^10^ This emphasizes that a more comprehensive understanding of the virus-induced manipulation of kinases can contribute not only to the identification of host factors per se but also to a better understanding of the pathophysiology. Ultimately, this knowledge may guide the development of novel antiviral strategies.

To date, there is limited information about the HEV infectious cycle, especially the usurpation of cellular host kinases. The recent identification of epidermal growth factor receptor (EGFR)^11^ as an HEV host factor has shed the first light on the virus-host dynamics of HEV. However, further analysis of the interaction between HEV and cellular kinases is desirable. To address this knowledge gap, we employed an RNAi mini screen targeting a selection of kinases, including mitogen-activated protein kinases, receptor tyrosine kinases (RTKs), and Src-family kinases (SFKs). Here, we report that HEV challenge triggered Yes1 kinase activity, which in turn was necessary for the efficient onset of HEV infection. Inhibiting Yes1 signaling restricted HEV multiplication across different cell culture models, including an HEV *in vivo* model system, highlighting its potential as a novel antiviral drug target.

## METHODS

### Cell culture

The human hepatoma cell line HepG2 (ATCC-Nr.: HB-8065) and the cell line Huh-7-S10-3 (a kind gift from Suzanne Emerson, NIH) was cultured in DMEM-high-glucose (Gibco, Cat. Nr. 11965) supplemented with 10% (vol/vol) fetal calf serum (Capricorn, Lot. Nr. CPC21-4114), 1% (vol/vol) non-essential amino acids (Gibco, Cat. Nr. 11140050), 100 IU/mL penicillin, 100 µg/mL streptomycin (Gibco, Cat. Nr. 15140), and 2 mM l-glutamine (Gibco, Cat. Nr. 25030) (=DMEM compl.). For virus titration and infection assays, the HepG2-subclone HepG2/C3A was cultured in Eagle’s minimum essential medium (Gibco, Cat. Nr.11095) supplemented with 10% (vol/vol) ultra-low IgG-fetal calf serum (Gibco, Cat. Nr. 16250-078, Lot 1939770), 100 μg/mL gentamicin (Gibco, Cat. Nr. 15710), 2 mM l-glutamine, 1 mM sodium pyruvate (Gibco, Cat. Nr. 11360), and 1% (vol/vol) non-essential amino acids (=minimum essential medium compl.). HepG2 and HepG2/C3A cells were grown on rat collagen-coated (SERVA Electrophoresis, Cat. Nr. 47256.01) cell culture dishes. Primary human hepatocytes (PHHs) were obtained from Primacyt as cryopreserved hepatocytes, and each donor was seeded according to the manufacturer’s instructions for the respective donor (for donor information, see Supplemental Table S1, http://links.lww.com/HC9/B60). PHHs were seeded on collagen-coated 24-well plates (Primacyt) and kept in a human hepatocyte maintenance medium (Primacyt). Patient informed consent was obtained by Primacyt, as stated on their website. All cells were kept at 37°C in a 5% (vol/vol) CO_2_ incubator.

### *In vitro* transcription and electroporation

Constructs of HEV Kernow/C1 p6 full length, Kernow/C1 p6 GFP, Kernow/C1 p6 gluc, Kernow/C1 p6 ΔORF3 are derived from Kernow/C1 p6 genome (GT3; GenBank Accession Nr. JQ679013) and 83-2 full length from HEV83-2-27 genome (GT3, GenBank Accession Nr. AB740232). RNA transcripts were produced through *in vitro* transcription. Kernow/C1 p6 constructs were linearized using *Mlu*I (New England Biolabs, Cat. Nr. R3198) and 83-2 constructs using *Hin*dIII (New England Biolabs, Cat. Nr. R3104). *In vitro* transcripts were produced as previously described.^12^,^13^ Transfection of *in vitro* transcripts into the respective cells was conducted through electroporation. In brief, 5 × 10^6^ cells in 400 μL cytomix containing 2 mM ATP (Cayman Chemical, Cat. Nr. 14498) and 5 mM glutathione (Sigma Aldrich, Cat. Nr. #G4251) were mixed with 5 µg *in vitro* transcript RNA and electroporated using the Gene Pulser System (BioRad). Electroporated cells were immediately resuspended in 12.1 mL DMEM compl., and the cell suspension was seeded on respective plates depending on the performed experiment: for luciferase assays, 2 × 10^4^ cells/well were seeded on a collagen-coated 96-well plate; for virus production, 12.5 mL were seeded on a collagen-coated 10 cm dish.

### Production of cell culture–derived HEV infectious particles

Cell culture-derived HEV (HEVcc) (Kernow/C1 p6, 83-2 and Kernow/C1 p6ΔORF3) was produced as previously described.^12^,^13^ Briefly, HEV *in vitro* transcripts were electroporated into HepG2 cells as described above. At 7 days post transfection, enveloped infectious HEVcc particles were harvested by filtering the supernatant through a 0.45 µM mesh (Filtropur 0.45, Sarstedt, Cat. Nr. 83.1826). The filtered supernatant containing enveloped HEVcc infectious particles (HEVcc env.) was stored at 4°C for up to 7 days. To harvest non-enveloped HEVcc infectious particles (HEVcc non-env.), the cells were trypsinized, neutralized with DMEM compl., and centrifuged at 500*g* for 5 minutes. The pellet was resuspended in 1.5 mL DMEM compl. and the cells were lysed through 3 freeze-thaw cycles in liquid nitrogen. Cell debris and the lysate were separated by centrifugation at 10,000*g* for 10 minutes. HEVcc non-env. was stored at -80°C until further usage. To determine the virus titer, the filtered supernatant containing HEVcc env. or the cell lysate containing HEVcc non-env. was titrated on HepG2/C3A cells and fixed and stained against ORF2 protein as as described in the supplementary method section (http://links.lww.com/HC9/B60) at 7 days post infection (p.i.). Focus forming units per mL (FFU/mL) were determined according to Todt et al.^13^ Please find more materials and methods in the Supplement, http://links.lww.com/HC9/B60.

## RESULTS

### RNAi mini screen identifies the kinase Yes1 as a novel host factor for HEV infection

**FIGURE 1:**
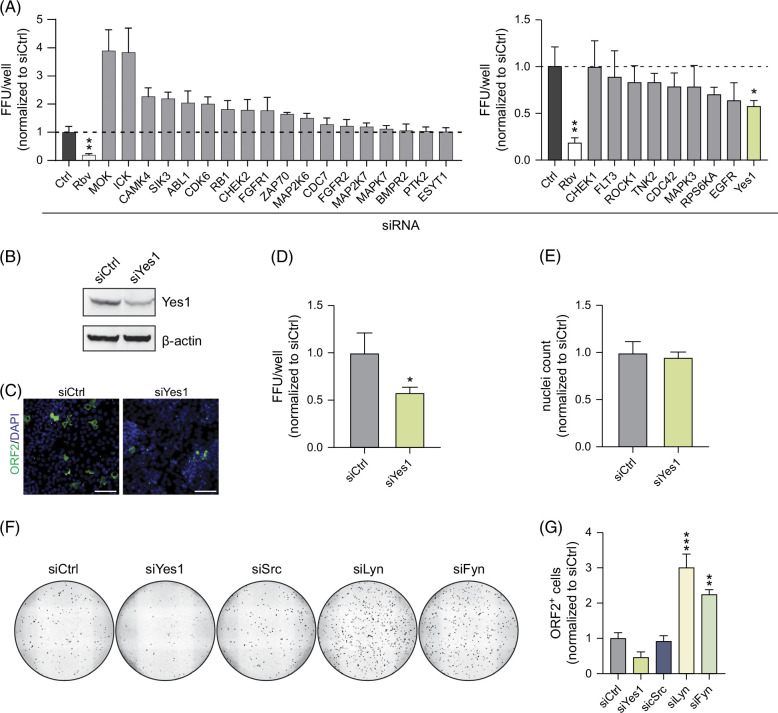
An siRNA mini screen identifies Yes1 as a novel host factor for HEV. (A) siRNA screen: HepG2 cells were transfected with the respective siRNA and infected with HEVcc p6 non-env. 2 days after transfection. FFU/well were counted after staining for the ORF2 protein at 5 days p.i. Counts were normalized to non-targeting control siRNA (siCtrl). Ribavirin (Rbv, 50 µM) served as efficient inhibition control and was applied simultaneously to infection. Left panel shows tested siRNAs that lead to an increase in observed HEV FFU/well, whereas the right panel depicts the ones that decreased HEV FFU/well. (B–E) Yes1 knockdown reduces HEV infections. (B) Yes1 expression in HepG2/C3A cells 2 days after transfection with siRNA targeting Yes1 or siCtrl, respectively, analyzed through western blot. (C) Representative fluorescent images and (D) quantification of HEV infections of siYes1- or siCtrl-transfected HepG2 cells infected with HEVcc p6 non-env. conducted like (A). (E) Cell viability of transfected cells from (C) was assessed by quantification of nuclei with CellProfiler. (F, G) Yes1 specifically is a novel HEV host factor. (F) Full well images of HEV infections (visible in black) of siYes1-, siSrc-, siLyn-, siFyn-, or siCtrl-transfected HepG2/C3A cells infected with HEVcc p6 non-env. Cells were infected 2 days after transfection and stained for ORF2 protein at 3 days p.i. Images were stitched and processed using Fiji. ORF2+ cells are indicated in black. (G) Quantification of HEV infections of siRNA-transfected HepG2/C3A cells infected with HEVcc p6 non-env. Cells were infected 2 days after transfection, and ORF2+ cells were determined at 3 days p.i. after staining for ORF2 protein and analysis with CellProfiler. All infection experiments were performed in triplicates. Mean and SEM are depicted from 3 independent experiments. Scale bars = 100 µm. To test statistical significance of mean differences, 1-way ANOVA followed by the Dunnett’s multiple comparison test (B, D) and Student’s t-test (C) were used. *p* values <0.05 (*), <0.01 (**), or <0.001 (***) were considered statistically significant. Only significant *p* values are shown. Abbreviations: FFU, focus forming units; HEVcc, cell culture–derived hepatitis E virus; p.i., post infection.

To identify signaling molecules influencing the HEV infectious cycle, we conducted an RNAi mini screen targeting a curated selection of kinases, including mitogen-activated protein kinases, RTKs, and SFKs (Figure 1A) and Supplemental Table S2, (http://links.lww.com/HC9/B60) CDC42^14^ and EGFR,^11^ 2 reported host factors of HEV, served as positive controls. We detected that knockdown of some kinases, including MOK and ICK, facilitated HEV infections (Figure 1A, left graph) while silencing of others, including the reported host factors CDC42 and EGFR, decreased HEV susceptibility (Figure 1A, right graph). Of note, we detected the strongest decrease of HEV susceptibility upon knockdown of Yes1, while the cell viability was unaffected (Figures 1B–E), a cytoplasmic non-receptor kinase belonging to the family of SFKs.^15^ Although members of the SFKs are reported to have highly conserved structures and overlapping functions,^16^,^17^ we did not observe a reduction in HEV infection events upon silencing of other SFKs including Src, Lyn, and Fyn (Figures 1F, G, Supplemental Figure S2A, http://links.lww.com/HC9/B60). By analyzing publicly available single-cell RNAseq data from liver tissue,^18^ we detected robust expression of Yes1 in hepatocytes (Supplemental Figure S1, http://links.lww.com/HC9/B60) and subsequently hypothesized that Yes1 is a potential host factor of HEV.

### Inhibition of Yes1 signaling restricts HEV infection

**FIGURE 2:**
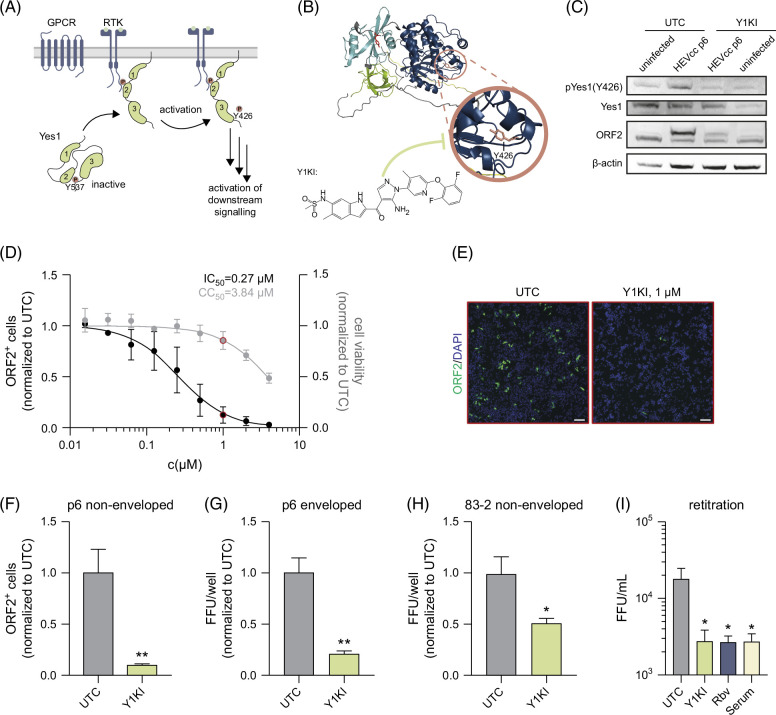
Yes1 activation is a novel HEV-host interaction, and a selective Yes1 kinase inhibitor can effectively reduce HEV infections. (A) Schematic illustration of Yes1’s activation. Yes1 kinase activity is inhibited by phosphorylation at Y537, leaving the protein in a clamped confirmation. Upon activation through GPCRs, RTKs, or Integrins through its SH2 or SH3 domain, Yes1 auto-phosphorylates at Y426 activating its kinase activity. (B) Structure of Yes1 predicted by AlphaFold and illustrated by Pymol. Blue: SH1; turquoise: SH2, green: SH3, red residue: Y537, and pink residue: Y426. Chemical structure of the Yes1 kinase inhibitor (Y1KI, CH6953755). (C) Expression of Yes1 and phosphorylation status at Y426 in HepG2/C3A cell lysates uninfected or infected with HEVcc p6 non-env. Three days p.i. of UTCs or treated with 1 µM Y1KI, respectively. (D, E) HEVcc p6 non-env. infection levels in HepG2/C3A cells under treatment with the indicated concentrations of Y1KI. (D) Quantification of HEV infection levels (black) at 3 days p.i. through CellProfiler analyzing ORF2 protein–positive cells (ORF2+) per image and cell viability (gray) measured using an MTT assay. (E) Representative fluorescence image of UTC or under treatment of 1 µM Y1KI, corresponding to the concentration used in (D) indicated by the red dot. (F–H) Quantification of HEV infection levels of HepG2/C3A cells treated with 1 µM Y1KI infected with HEVcc p6 non-env. (F), HEVcc p6 env. (G), or HEVcc 83-2 non-env. (H). (I) Quantification of intracellular viral titers recovered from lysed HepG2/C3A cells at 3 days p.i. with HEVcc p6 non-env. under treatment of 1 µM Y1KI, 50 µM Rbv, or 1:50 of anti-HEV serum. For all graphs, mean values from 3 independent experiments are depicted. Dose-dependent treatment and 50% inhibitory concentrations (IC_50_ and CC_50_) were calculated employing a 4-parameter log-logistic nonlinear regression model (D). To test the significance of mean differences, One-way ANOVA, followed by the Dunnett’s multiple comparison test (I) or Student’s t-test (F–H) were used. *p* values <0.05 (*), <0.01 (**), <0.001. p values >0.05 were considered to be not significant. Scale bars = 100 µM. Abbreviations: FFU/mL, focus forming units per mL; GPCR, G-protein–coupled receptors; HEVcc, cell culture–derived hepatitis E virus; MTT, 3-(4,5-dimethylthiazol-2-yl)-2,5-diphenyltetrazolium bromide; p.i., post infection; RTK, receptor tyrosine kinases; UTC, untreated control cells.

Given that Yes1 is a central node in cellular signaling, we next asked whether its signaling activity may be altered upon HEV infection. A hallmark for the activation of the Yes1 kinase activity and its downstream signaling is the phosphorylation of Y426 by upstream RTKs, G-protein-coupled receptors or integrins (Figure 2A). Of note, integrin α3^19^ and EGFR^11^ (an RTK) both have recently been shown to be involved in HEV entry. To test whether HEV infection modulates the kinase activity of Yes1, we aimed to assess the expression of Yes1 and its phosphorylation at Y426 through western blot analysis in the presence and absence of HEV infection. As a control, we used a Yes1-specific kinase inhibitor (CH6953755,^20^ in the following referred to as Y1KI) that inhibits the phosphorylation of Yes1 at Tyr426 (Figure 2B). In these experiments, we detected increased levels of Y426-phosphorylation of Yes1 upon HEVcc p6 non-env. infection compared to uninfected cells (Figure 2C, Supplemental Figures S2C, D, http://links.lww.com/HC9/B60), suggesting that HEV modulates both activity and signaling of Yes1. Notably, when we treated cells with Y1KI, we observed an ablation of HEV-induced phosphorylation of Yes1 concomitant with reduced levels of HEV-ORF2, indicating that phosphorylation of Yes1 is critical for the viral life cycle progression.

To characterize the antiviral activity of Y1KI, we next performed HEV infection experiments in the presence of the inhibitor. Here, we detected a dose-dependent inhibition of HEV life cycle progression with a calculated 50% inhibitory concentration (IC_50_) of 0.27 µM, while the cell viability was not affected at these concentrations with a 50% cytotoxic concentration (CC_50_) of 3.84 µM (Figure 2D, respective full well images in Supplemental Figure S3A, http://links.lww.com/HC9/B60). Next, we asked whether the inhibition of Yes1 activity is capable of restricting different forms (non-enveloped and enveloped HEV) as well as different isolates of HEV (p6 and 83-2, as well as rat HEV LA-B350). In subsequent infection experiments, we detected a significant restriction for all tested variants, indicating that Yes1 is a critical host factor for the propagation of different forms and isolates of HEV (Figures 2F–H, Supplemental Figure S6A, http://links.lww.com/HC9/B60, respective full well images in Supplemental Figures S3B–D, http://links.lww.com/HC9/B60). To further validate the importance of Yes1 during HEV infection, we quantified the production of progeny virus particles of HEV-infected cells in the presence and absence of Y1KI. Ribavirin and anti-HEV serum served as controls. Here, we observed that treatment with Y1KI decreased infectious titers of progeny virus by about 1 order of magnitude (Figure 2I).

Taken together, we could show that inhibition of Yes1 phosphorylation by Y1KI leads to a potent inhibition of HEV infection of different HEV isolates. These data suggest that Yes1 kinase activity is critical for robust HEV infection, and its inhibition serves as an effective antiviral strategy *in vitro*.

### Yes1 activation is important for the early onset of HEV infection

**FIGURE 3:**
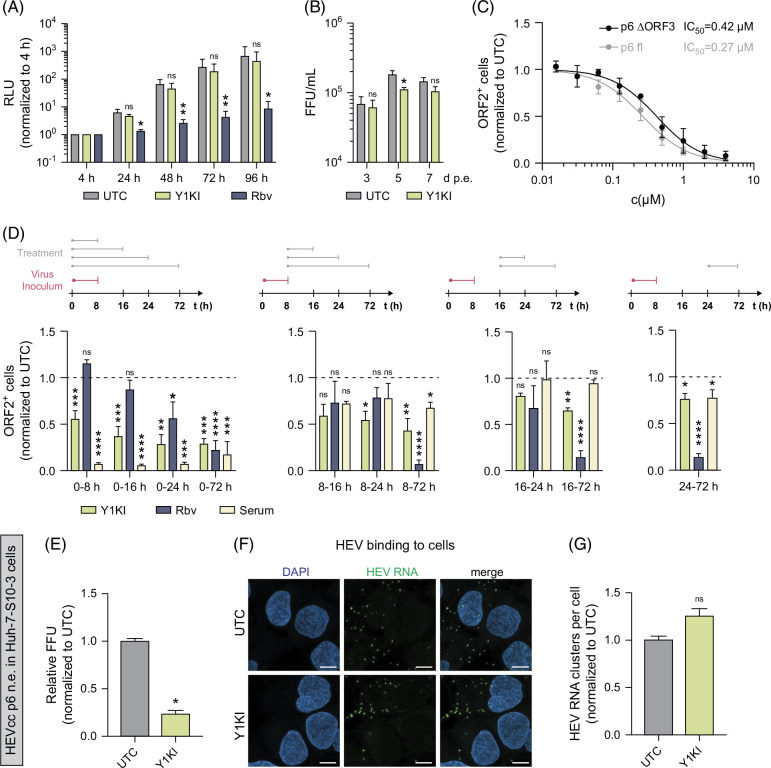
Y1KI affects HEV’s early onset of infection independently of ORF3. (A) HEV replication levels in HEV p6 SGR system in HepG2/C3A cells under treatment of 1 µM Y1KI or 50 µM Rbv at the indicated time points. (B) Viral titers of HEVcc p6 non-env. produced after electroporation of HEV p6 RNA into HepG2/C3A cells and treatment with 1 µM Y1KI, thereby excluding entry. (C) HEVcc p6 ΔORF3 non-env. (black) versus HEVcc p6 non-env. (gray) infection in HepG2/C3A cells under treatment with the indicated concentrations of Y1KI fixed at 3 days p.i. (D) Time-of-addition assay. HepG2/C3A cells were infected with HEVcc p6 non-env. for 8 hours. Inoculum was removed, and cells were washed thrice with PBS. Cells were treated with 1 µM Y1KI, 50 µM Rbv, or anti-HEV serum (1:50), respectively, for the duration of the indicated time points. Cells were stained for ORF2 protein at 3 days p.i. and HEV infection levels were quantified. (E) Quantification of HEV infection levels of Huh7-S10-3 cells treated with 1 µM Y1KI infected with HEVcc p6 non-env. (F, G) RNA FISH of HEV RNA during binding of HEV. Huh-7-S10-3 cells were treated with 1 µM Y1KI and prechilled on ice for 10 minutes before inoculation with HEVcc p6 non-env. Following infection on ice, the cells were incubated at 4°C for 2 hours to allow particle binding but not internalization. The cells were fixed, nuclei stained with DAPI, and HEV genomes detected by RNA-FISH. (F) Images represent single slices of confocal images acquired on a Leica SP8 confocal microscope. Scale bars = 10 µm. (G) The detected HEV RNA clusters were quantified using CellProfiler. HEV particles per cell (nuclei) were calculated by dividing the total number of detected HEV RNA clusters by the number of nuclei in an image frame and 1–6 random image frames containing 6–18 nuclei per image. For all graphs, mean and SEM from at least 3 independent experiments performed in triplicates (A–D) are depicted. Dose-dependent treatment and 50% inhibitory concentrations (IC_50_) were calculated employing a four-parameter log-logistic nonlinear regression model (C). To test significance of mean differences, Two-way ANOVA followed by the Šídák multiple comparison test (A, B), 1-way ANOVA followed by the Dunnett’s comparison test (D), or Student’s t-test (E, G) were used. *p* values <0.05 (*), <0.01 (**), <0.001 (***), and <0.0001 (****). *p* values >0.05 were considered to be not significant (n.s.). Abbreviations: FISH, fluorescence in situ hybridization; HEVcc, cell culture–derived hepatitis E virus; p.i., post infection; SGR, subgenomic replicon.

Having identified that Yes1 kinase inhibition restricts the HEV infectious cycle, we next aimed to understand which life cycle step Y1KI conveys restriction of HEV infection. To investigate whether perturbation of Yes1 activation affects HEV replication, we used the HEV subgenomic replicon system that carries a *Gaussia* luciferase reporter (HEV Gluc),^21^ enabling to monitor HEV RNA replication kinetics. Using the HEV Gluc subgenomic replicon system, no significant effect of Y1KI treatment on HEV RNA replication levels was observable (Figure 3A). Furthermore, dose-dependent reduction in replication levels at 72 hour p.i. aligned with cell cytotoxicity and resulted in similar IC_50_ and CC_50_ values (Supplemental Figure S4C, http://links.lww.com/HC9/B60), further indicating no measurable effect of Yes1 inhibition on HEV replication in this assay.

To investigate whether Yes1 affects progeny virus production, we next transfected HEV p6 full-length RNA into hepatoma cells in the presence or absence of Y1KI, followed by titration on HepG2/C3A cells. Thereby, we found no difference in viral titers harvested 3 and 7 days post-transfection and only a modest yet significant decrease when harvested 5 days post-transfection (Figure 3B), suggesting that Yes1 signaling is dispensable for late life cycle steps, such as assembly of HEV particles.

Notably, the HEV ORF3 protein was reported to interact with the SH3 domain of the SFK Src.^22^ Given that Yes1 shares homology with Src and harbors an SH3 domain, we asked whether Yes1 inhibition is ORF3-dependent, which in turn may cause the restriction of HEV infection. To test this hypothesis, we used an HEV p6 virus lacking ORF3 (HEV p6 ΔORF3 non-env.) for infection experiments. This allowed us to exclude any interactions of Yes1 and HEV ORF3. Of note, we found that Y1KI was able to restrict HEVcc p6 ΔORF3 non-env. infections in a similar dose-dependent manner as for HEV variant harboring ORF3 (Figure 3C, full well images in Supplemental Figures S4A, B, http://links.lww.com/HC9/B60). These data suggest that inhibition of Yes1 activity does not mediate its antiviral effect through interplay with HEV ORF3.

To further reveal which HEV life cycle step is affected by perturbation of Yes1 activation, we performed time-of-addition infection assays. The HEV polymerase inhibitor and nucleoside analog Rbv served as a control for replication inhibition, whereas anti-HEV serum served as a control for attachment and entry. While early treatment with Y1KI potently restricted HEV infection, Yes1 inhibition at late time points did not show similar effects (Figure 3D). Since we also detected that inhibition of Yes1-kinase activity by Y1KI reduced HEV infections even after removal of the HEV inoculum (Figure 3D, middle panels), our data suggest an important role of Yes1-kinase activity during the early onset of infection of HEV.

Further analysis of HEV’s entry and early onset of infection was performed with a binding experiment assessed through fluorescent in situ hybridization^23^ (Figures 3E–G). Here, we detected no effect of Yes1 kinase inhibition on the binding process of HEV. In summary, our data indicate that the activity and signaling of Yes1 are needed for the efficient early onset of infection, excluding the initial binding of HEV particles. This suggests a novel and previously unknown role of the tyrosine kinase Yes1.

### Yes1 inhibition restricts HEV infections in PHHs *ex vivo* and in rats *in vivo*

Considering that anti-HEV therapy is currently limited to off-label use of Rbv, where resistance mutations to Rbv treatment have been described,^24^,^25^ and knowledge about HEV pathogenesis is scarce, we aimed to evaluate whether Y1KI might be considered as an add-on during Rbv treatment. As a proof of principle, we therefore performed combinatory treatment of Y1KI and Rbv in different doses in HepG2/C3A cells (Supplemental Figure S5A, http://links.lww.com/HC9/B60). Using the Loewe synergy score, we did not find considerable antagonistic effects of Y1KI and Rbv co-treatment (Supplemental Figure S5B, http://links.lww.com/HC9/B60). Of note, the absence of an antagonistic effect indicates that Y1KI could potentially be used as combined therapy with Rbv. Given that SFKs, and potentially Yes1, contribute to the signal transmission of the HEV host factor EGFR, we also performed the combinatory treatment with the EGFR kinase inhibitor Erlotinib and Y1KI. Here, we detected additive effects (Supplemental Figures S5C, D, http://links.lww.com/HC9/B60), which could imply 2 distinct mechanisms of how EGFR and Yes1 are involved as host factors for HEV but need further investigation. Overall, these data support the hypothesis that Y1KI restricts HEV through mechanisms other than Rbv and Erlotinib.

**FIGURE 4:**
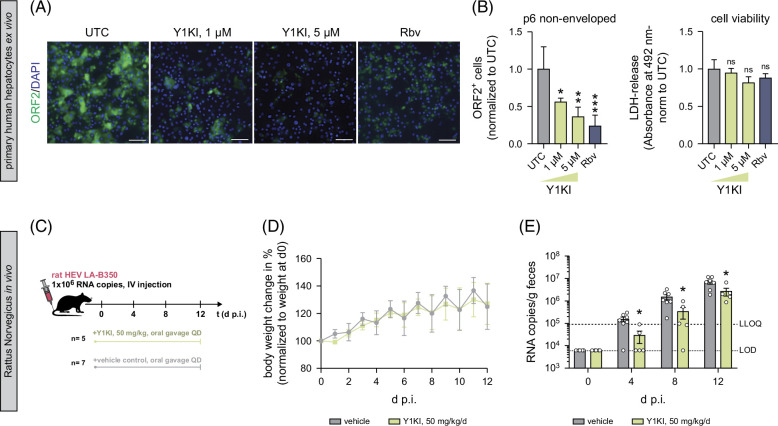
Y1KI restricts HEV infections in PHHs and ratHEV infections *in vivo* in rats. (A) Representative immunofluorescence images of HEVcc p6 non-env. infected PHHs at 3 days p.i. under treatment of UTC (n = 5), 1 µM (n = 5), or 5 µM Y1KI (n = 3), and 25 µM Rbv (n = 5), respectively. (B) Quantification of HEV infection levels (left) and cell viability (right) measured using an LDH-release assay at 3 days p.i. and normalized to untreated cells. (C) Schematic *in vivo* experimental procedure: *Rattus norvegicus* was treated with either vehicle or 50 mg/kg Y1KI 2 h before infection with rat HEV LA-B350 through i.v. injection into the tail vein. Daily treatment (QD) continued up to 12 days p.i. and fecal samples were taken every 4 days. At 12 days p.i., rats were sacrificed. Data presented are from 2 independent studies, with a total n = 7 in the vehicle group and n = 5 in the Y1KI-treated group. (D) Average percent body weight change of rats normalized to the respective weight at 0 days p.i. (E) Rat HEV RNA copy numbers in fecal samples of rats treated with 50 mg/kg/d Y1KI or vehicle measured through RT-qPCR. For graphs in (B), mean values from at least 3 independent experiments are depicted. Mean values (D) and all tested values of all rats (E) are shown. To test the significance of mean differences, either the Student’s t-test (E) or 1-way ANOVA, followed by the Dunnett’s comparison test (B), was used. *p* values <0.05 (*), <0.01 (**), and <0.001 (***). *p* values >0.05 were considered to be not significant (n.s.). Scale bars = 100 µM. Abbreviations: HEVcc, cell culture–derived hepatitis E virus; i.v., intravenous; LDH, lactate dehydrogenase; LLOQ, lower limit of quantification; LOD, limit of detection; PHH, primary human hepatocyte; p.i., post infection; QD, once daily; UTC, untreated control cells.

Intending to assess the application of Y1KI in a more authentic cell culture model, we used PHHs *ex vivo*. PHHs retain high liver-specific functionality, making them valuable and important cell culture models for studying liver-specific processes, drug metabolism, and viral infections. We treated PHH cultures with Y1KI upon HEV infection and observed that Y1KI restricted HEV infection in a dose-dependent manner, while cell viability assessed through lactate dehydrogenase levels in the supernatant remained unchanged compared to untreated PHHs (Figures 4A, B).

Finally, we evaluated the antiviral potential of Y1KI in an *in vivo* model using athymic nude rats infected with rat HEV LA-B350.^26^,^27^ Rats were treated once daily (QD) with 50 mg/kg Y1KI or vehicle control for 12 days with the start of treatment 2 hours before infection with rat HEV (Figure 4C). No adverse effects or significant weight loss were observed (Figure 4D), showing that the administered dose was safe for the rats. Daily treatment of Y1KI resulted in a moderate but significant reduction in rat HEV RNA in fecal samples on days 4, 8, and 12 p.i. (Figure 4E). Lower viral RNA levels were also present in the liver, spleen, and intestine on 12 days p.i., although they did not differ significantly from those in the vehicle-treated rats (Supplemental Figures S6B–F, http://links.lww.com/HC9/B60). No difference in ratHEV RNA levels was detected in serum, but due to the overall considerably lower ratHEV RNA levels in serum compared to the other tissues tested, serum ratHEV levels in rats might not represent a robust indicator for viremia. The data hence prove the role of Yes1 as a host factor needed for efficient HEV infection in an authentic *in vivo* system and additionally highlights the potential of host factor targeted antivirals.

Taken together, our data demonstrate that Y1KI restricts HEV infection in primary cells *ex vivo* and in rats *in vivo*, proving the principal role of Yes1 as a critical host factor needed for efficient HEV onset of infection.

## DISCUSSION

Although HEV is one of the most common causes of viral hepatitis worldwide, it remains an underinvestigated health burden. To identify signaling molecules that impact the infectious cycle of HEV, we carried out an RNAi mini screen, targeting kinases from diverse families, including mitogen-activated protein kinases, RTKs, and SFKs. Here, we identified a dependence of HEV infection on the signaling molecule Yes1, a cytoplasmic non-RTK and member of SFKs.^15^ Since the knockdown of Yes1 showed the strongest phenotype among all tested kinases and given its robust expression in liver tissues,^18^ we hypothesized that Yes1 is a host factor for HEV. Despite SFK members having been suggested to have redundant functions^16^ and considering that gene-editing of Yes1 can be partially compensated by other SFK members,^17^ we could reveal that the decrease in HEV susceptibility upon knockdown was specific for Yes1. This indicates that a particular function of Yes1 is critical for HEV’s infectious cycle.

Yes1 is structurally divided into 4 Src-homology domains (SH1–4) that are highly conserved among the members of SFKs.^28^ myristoylations in the SH4 domain anchor Yes1 to the cellular membranes where it can interact with RTKs like EGFR, G-protein–coupled receptors and cytokine receptors like Integrins, conveying phosphorylations through its SH2 and SH3 domains.^29^ An activation of Yes1 by upstream kinases is passed forward by phosphorylation of Tyr426, triggering the kinase function of Yes1 and phosphorylation of downstream proteins, like the Yes-associated protein.^30^ Yes1 is, therefore, a central node in the cellular signal transduction and involved in numerous signaling pathways leading to cell proliferation and survival.^29^ In this study, we found that HEV infection causes an increase in phosphorylation of Yes1 at Tyr426, thus leading to the activation of its downstream signaling. Considering the high conservation of the structure and phosphorylation sites of SFKs, we employed a chemical kinase inhibitor (CH6953755, Y1KI) reported to be specific to Yes1 compared to other broad-spectrum SFK inhibitors.^20^,^29^ Notably, inhibition of Yes1 signaling by Y1KI led to a ~20-fold reduction in HEV infections for both enveloped and non-enveloped viral HEV particles, highlighting the importance of Yes1’s signaling for HEV infections.

Previous studies have described SFKs, including Yes1, as viral host factors with varying mechanisms of action. Specifically, interactions of SFKs have been studied in the context of HCV infections: direct interaction with the SH2 and SH3 domains of Src was reported to support the complex formation of NS5A and NS5B, which is required for successful viral replication.^31^ Furthermore, an involvement of Yes1 in West Nile Virus particle maturation was discovered, and Yes1 proposed to play a role in West Nile Virus glycoprotein trafficking from the endoplasmic reticulum to post–endoplasmic reticulum compartments.^32^ Also in the context of HEV, a study by Korkaya et al^22^ found an interaction of the HEV ORF3 protein with the SH3 domain of the SFK-member Src. Even though Yes1 and Src have—despite their similar structure—unique roles,^16^,^17^ we investigated a potential interplay of HEV ORF3 and Yes1 by using an HEV construct lacking HEV ORF3. The HEV ORF3-encoded protein has been reported to be essential for viral egress but not for assembly.^33^,^34^ Here, we found Yes1’s mode of action to be independent of HEV ORF3 and not involved in viral egress, hence revealing a novel mechanism of SFK-HEV interplay and a novel and specific role for Yes1. Strikingly, by using time-of-addition assays, we found Yes1’s signaling to restrict HEV’s early onset of infection without affecting HEV binding. We, therefore, propose Yes1’s signaling to be a critical factor for HEV pathogenesis by creating an optimal environment for the viral onset of infection. Consequently, this raises the question of how activated Yes1 signaling can favor HEV infection. It is reported that Yes1 integrates and forwards information from different receptors that are located on the cell surface, including the HEV host factors integrin α3^19^ and EGFR.^11^ Thus, it is tempting to speculate whether the activation of Yes1 signaling is based on an interplay with 1 of these 2 factors.

For multiple viruses, evidence suggests that they can use the EGFR endocytic machinery or that EGFR can act as a co-receptor to stabilize the interaction of virus particles to their cognate entry receptor.^35^,^36^ Considering that EGFR’s proviral effect for HEV is independent of its canonical signaling and kinase activity^11^ and given that combinatory treatment of Y1KI and the EGFR kinase inhibitor erlotinib indicated an additive effect, we hypothesize that EGFR signaling is not a trigger for activation of Yes1 signaling in the HEV infection setting. In addition to EGFR, Yes1’s kinase activity can be activated by integrins.^37^ Binding to integrins can trigger an outside-in signaling, leading to a rearrangement of the actin-network among other changes through downstream signaling cascades enabling endocytosis of the virus.^38^ SFKs, and as such Yes1, are involved in these signaling networks. Yes1’s kinase activity could, therefore, potentially contribute to the entry of HEV. Future studies have to investigate the interplay of Yes1 and involved receptors to detangle the process of HEV early onset of infections and whether Yes1 could possibly be directly interacting with viral proteins since currently further experiments are limited due to the few available assays.

Taken together, we identified the dependence of HEV on Yes1 signaling, which may contribute to the so far scarce knowledge of HEV’s pathogenesis in the future. Moreover, we provide Y1KI as a novel antiviral drug specifically targeting an HEV host factor. Of note, we validated the inhibitory effect of the Yes1 kinase inhibitor (Y1KI) *ex vivo* in PHHs and found an antiviral trend of Y1KI *in vivo* in a rat HEV model system. These data not only prove and underline the relevance of this factor but also highlight the potential of host factor-targeted antivirals presenting a candidate antiviral treatment option to tackle HEV.

## Supplementary Material
