# Supplementary material for: The tyrosine kinase Yes1 is a druggable host factor of HEV
Source: Hepatol Commun. 2024 Oct 17;8(11):e0553. doi: 10.1097/HC9.0000000000000553 (PMC11495762; doi:10.1097/HC9.0000000000000553)
Supplement: Supplementary file 1 [file hc9-8-e0553-s001.docx]

**HEP4-24-0647 - The tyrosine kinase Yes1 is a druggable host factor of hepatitis E virus**

Jil Alexandra Haase^1^, Abarna Baheerathan^2^, Xin Zhang^3^, Rebecca Menhua Fu^4,5^, Maximilian K. Nocke^1^, Charlotte Decker^4,5^, Viet Loan Dao Thi^4,6^, Daniel Todt^1,7^, Johan Neyts^3^, Suzanne J. F. Kaptein^3^, Eike Steinmann^1,8#^, Volker Kinast^1,9#^

^1^ Department of Molecular and Medical Virology, Faculty of Medicine, Ruhr University Bochum, Bochum, Germany

^2^ Institute of Clinical and Molecular Virology, University Hospital Erlangen, Friedrich-Alexander-University Erlangen-Nürnberg, Erlangen, Germany

^3^ KU Leuven Department of Microbiology, Immunology and Transplantation, Rega Institute for Medical Research, Laboratory of Virology and Chemotherapy, Leuven, Belgium

^4^ Schaller Research Group, Department of Infectious Diseases and Virology, Heidelberg University Hospital, Heidelberg, Germany

^5^ Heidelberg Biosciences International Graduate School, Heidelberg University, Heidelberg, Germany

^6^ German Centre for Infection Research (DZIF), Partner Site Heidelberg, Heidelberg, Germany

^7^ European Virus Bioinformatics Centre (EVBC), Jena, Germany

^8^ German Centre for Infection Research (DZIF), External Partner Site, Bochum, Germany

^9^ Department of Medical Microbiology and Virology, Carl von Ossietzky University Oldenburg, Oldenburg, Germany

**Supplementary Materials and Methods:**

siRNA-Mediated Knockdown

Two short interfering RNAs (siRNA) per gene were used (see Supplementary Table 2) and a control siRNA (Ambion/ThermoFisher, ID: s814). The two siRNAs per gene were pooled and 0.5 µM of each transfected into HepG2/C3A cells using Lipofectamine RNAiMax (Invitrogen, Cat. Nr. 13778) following the manufacturer’s instructions. Two days post transfection, the cells were either lysed for western blot analysis (described below) or infected with HEVcc p6 non-env. (MOI 0.1–1). Cells were subjected to immunofluorescence analysis 5 d p.i. (as described below) staining ORF2 protein. FFU/well were counted using Fiji-ImageJ or ORF2 protein positive (ORF2+) cells per image section (4× magnification for HepG2/C3As and 10× magnification for PHHs, randomly taken) determined with the help of CellProfiler.

HEV Infection Assays with Non-Enveloped HEVcc

1×10^4^ HepG2/C3A cells were seeded on collagen-coated 96-well plates one day prior to infection with a multiplicity of infection (MOI) of 0.1 – 2. PHHs were plated as described above one day prior to infection with a MOI of 0.1 – 0.2. Yes1 kinase inhibitor (Y1KI, CH6953755, MedChemExpress, Cat. Nr. HY-135299) of the indicated concentration (solved in DMSO) was applied simultaneously with the virus inoculum. The medium of infected PHHs was changed at approximately 16 h p.i. to HHMM culture medium (Primacyt, Cat. Nr. HHMM-500). Cells were fixed at 3 d p.i. using 3% Paraformaldehyde (PFA) and subjected to immunofluorescent staining of ORF2 protein as described below. Either FFUs/well were counted or ORF2 protein positive (ORF2+) cells per image section (4× magnification for HepG2/C3As and 10× magnification for PHHs, randomly taken) determined with the help of CellProfiler. Infection experiments with HEVcc p6 non-env. in Huh-7-S10-3 cells were carried out as previously described ^1^. Infection experiments of rat HEV LA-B350 liver homogenate in Huh7 cells were performed as previously described ^2^.

HEV Infection Assays with Enveloped HEVcc

1×10^4^ HepG2/C3A cells were seeded on collagen-coated 96-well plates one day prior to infection with 200 µL (approx. MOI 0.02 – 0.05) of enveloped HEVcc. Y1KI (1 µM, solved in DMSO) was applied to the cells simultaneously with virus inoculation. After 24 h p.i. the inoculum was removed and the medium replenished and the Y1KI renewed. Cells were fixed at 3 d p.i. using 3% PFA and subjected to immunofluorescent staining of ORF2 protein as described below. FFUs/well were determined.

Time-of-Addition Assay

1×10^4^ HepG2/C3A cells were seeded on collagen-coated 96-well plates one day prior to infection with HEVcc p6 non-env. (MOI 0.1 – 1) at timepoint 0 h p.i. For the indicated conditions, 1 µM Y1KI, 50 µM Ribavirin (Rbv, Sigma-Aldrich, Cat. Nr. R9644) or anti-HEV Serum (1:50) were applied simultaneously to infection. For all conditions, the inoculum was removed at 8 h p.i. and the cells carefully washed twice with PBS before fresh MEM compl. was added with the indicated supplement (DMSO, Y1KI, Rbv or anti-HEV Serum). For the indicated conditions, the medium containing the supplement was removed at timepoints 16 h p.i. and 24 h p.i. and the cells carefully washed twice with PBS before addition of MEM compl. without any supplements. Cells were fixed at 3 d p.i. using 3% PFA and subjected to immunofluorescent staining of ORF2 protein as described below. ORF2+ cells per image section were determined with the help of CellProfiler.

HEV Luciferase Replication Assays

HEV replication was monitored using a Gaussia luciferase (Gluc) construct ^3^ replacing the ORF2 in the HEV Kernow/C1 p6 genome (GeneBank Accession number: JQ679013). Cells electroporated with HEV p6 Gluc RNA were seeded as described above and treated with either DMSO, Rbv (50 µM), or Y1KI of the indicated concentrations. Supernatant was taken at timepoints 4, 24, 48, 72 and 96 h p.e (kinetic) or 72 h p.i. (dose-response). To measure the luciferase activity, 20 μL of the collected supernatant was transferred to a white, flat-bottom microplate (Greiner Bio-One, Cat. Nr. 655074). The supernatant was subsequently incubated with luciferase substrate (1 μmol/L of coelenterazin in PBS, Carl Roth, Cat. Nr. 4094.3) and luciferase activity was measured in a luminometer (CentroXS3 LB960, Berthold technologies).

HEV Retitration

1×10^5^ HepG2/C3A cells/well were seeded on a collagen-coated 12-well plate one day prior to infection with HEVcc p6 non-env. (MOI 0.1) and simultaneous treatment with either DMSO, Y1KI (1 µM), Rbv (50 µM) or anti-HEV serum (1:50). The inoculum was removed 24 h p.i. and fresh MEM compl. with the indicated supplements replenished. Intracellular virus was harvested on days 3, 5 and 7 p.i. as described above with the following change: the cell pellet was resuspended in 100 µL per well and two wells per condition were used. Viral titers were determined in duplicates as described above.

Immunofluorescence Staining and Microscopy

Prior to intracellular immunofluorescent staining, cells were fixed with 3% paraformaldehyde (PFA, Roth, Cat. Nr. 93351) for a minimum of 10 min and permeabilized using 0.1% Triton X-100 (Carl Roth, Cat. Nr. 3051.3) in PBS for 5 min. The cells were then blocked in 5% horse-serum (Gibco, Cat. Nr. 26050-088) in PBS for at least 1 h. HEV infections were visualized using a polyclonal HEV genotype-3 capsid (ORF2) protein-specific rabbit hyperimmune serum (diluted 1:5 000 in 5% horse serum, kindly gifted by Prof. Rainer G. Ulrich, Friedrich Loeffler Institute, Germany). Cells with the respective antibody were incubated overnight at 4 °C on a rocking shaker. Unbound primary antibody was washed away with PBS thrice and the cells incubated with secondary antibody (goat anti-rabbit AlexaFluor 488, 1:1 000 in 5% horse-serum, Invitrogen, Cat. Nr. A-11008) for 2 h at room temperature on a rocking shaker in the dark. Unbound antibody was washed away twice with PBS before DNA staining with 4′,6′-175 diamidino-2-phenylindole (DAPI, Invitrogen, Cat. Nr. D1306, 1:10 000 in H2O) for 5 min. Cells were washed twice with PBS again and stored in water at 4 °C in the dark until imaging. All steps were performed at room temperature unless stated otherwise. Images of fluorescently stained cells were taken with a Keyence 179 BZX800 microscope with 4×, 10× or 20× objectives.

Binding and internalization assays

One day after seeding 3×10^4^ Huh-7-S10-3 cells in a well of a 48-well plate, Huh-7-S10-3 cells were treated with the respective chemical inhibitor and pre-chilled on ice for 10 min. The cells were then inoculated with HEVcc p6 non-env. (MOI=30 genome equivalents (GE)/cell, as previously described ^1^) and incubated for 2 h to allow particle binding. The cells were then fixed, stained with DAPI and HEV RNA was detected by RNA fluorescent in situ hybridization (FISH) (version 2 kit) using the ORF1 probe (see below).

In situ labeling of viral RNA and immunofluorescence staining

For the detection of HEV RNA the RNAscope® Fluorescent Multiplex Kit version 2 (ACDBio) was used. Infected Huh-7-S10-3 cells seeded onto coverslips were fixed in 4% PFA and permeabilized in 0.1% Trion-X100, followed by H_2_O_2_ treatment for 10 min at RT. The positive strand of HEV RNA was targeted by the ORF1 probe (ACDBio, Cat No. 579831). The coverslips were mounted using the ProLong™ Glass Antifade Mountant (Thermo Fisher Scientific) and cured for at least 24 h in the dark.

Confocal microscopy and image analysis of RNA FISH

Multichannel z-series with a z-spacing of 10 μm, or single slice confocal images were acquired using a Zeiss Airyscan LSM900 confocal microscope, as indicated in the figure legend. A 63× oil immersion objective was used for all images. For quantification of HEV genomes per cell during binding and internalization, maximum projections of full z-series were used. The genomes per cell were estimated by dividing the total number of detected genomes (RNA clusters) by the number of nuclei in a frame. Images were processed using the Zen software and inspected manually before quantifications using CellProfiler.

Western (Phospho-) Blot

For western (phospho-) blot analysis, cells were washed with TBS once and lysed with M-per buffer (Thermo Scientific, Cat. Nr. 78501) supplemented with Pierce Protease Inhibitor Mix (Thermo Scientific, Cat. Nr. A32953) and 1×Phosphatase inhibitor mix II and III (MedChemExpress, Cat. Nr. HY-K0022 and HY-K0023, respectively only for phospho-blots). Cells were frozen once at -80 °C before centrifugation at 10 000×g for 15 min. The supernatant was supplemented with Laemmli-sample buffer to a final concentration of 1× and the sample further denatured at 95 °C for 5 min and resolved by SDS-PAGE before being transferred to nitrocellulose membranes by wet tank blotting. For size comparison the PageRuler Plus Prestained Protein Ladder (Thermo Fisher Scientific, Cat. Nr. 26619) was used. Membranes were blocked for 1 h on a rocking shaker using 5% milk in TBS containing 0.05% Tween20 (TBS-T). Subsequently, the blots were incubated with primary antibody diluted in 0.5% milk-TBS-T overnight at 4 °C on a rocking shaker. In order to visualize total protein of SFK members, following antibodies were used: Yes1: a polyclonal rabbit antibody (CST, Cat. Nr. 3201, 1:1 000), Src: monoclonal rabbit antibody (CST, Cat. Nr. 2109, 1:1 000), Lyn: monoclonal rabbit antibody (CST, Cat. Nr. 2796, 1:1 000), Fyn: polyclonal rabbit antibody (CST, Cat. Nr. 4023, 1:1 000) and ORF2: see immunofluorescence staining section (1:1 000). To stain phosphorylated-Yes1 at Tyr426 the polyclonal p-SFK antibody (CST, Cat. Nr. 2101, 1:1 000) was used, followed by washing with TBS-T thrice and incubation with secondary horseradish peroxidase (HRP) conjugated polyclonal goat anti-rabbit antibody (Abcam, Cat. Nr. #ab97051, 1:10 000) for 2 h at room temperature. In order to visualize the housekeeping protein β-Actin, a primary antibody targeting β-Actin was used, which was already conjugated with HRP (Sigma-Aldrich, Cat. Nr. A3854, 1:10 000) and the blot incubated with it for only 2 h at room temperature on a rocking shaker. The membranes were washed twice with TBS-T and once with TBS before being developed using the Pierce ECL Western Blotting Substrate (Thermo Scientific, Cat. Nr. 32109) and analyzed using a chemiluminescence imaging machine (Celvin S 420, Biostep). Reblotting was done using the mild ReBlot antibody stripping buffer (Merck, Cat. Nr.2502) according to the manufacturer’s instructions. Blots were washed twice with TBS-T before starting with the blocking step and before incubation with the next antibody.

Cell Viability and Cytotoxicity Assays

To assess the cell viability upon siRNA-mediated Yes1 knockdown, the DAPI stained nuclei (see above) were automatically counted with the help of CellProfiler in images taken 5 days post transfection.

To determine the cell viability upon treatment with Y1KI, an MTT (3-(4,5-dimethylthiazol-2-yl)-2,5-diphenyltetrazolium bromide) assay (HepG2/C3As) was performed or the cell cytotoxicity determined using the LDH-assay (Promega. Cat. Nr. G1780) (PHHs).

For MTT assay, 1×10^4^ HepG2/C3A cells were seeded on a collagen-coated 96-well plate one day prior to treatment with the indicated amounts of Y1KI. Three days post treatment, the cells were incubated with MTT substrate (0.5 mg/mL, Biomol, Cat. Nr. 15655) supplied in MEM compl. at 37 °C in a 5% (v/v) CO_2_ incubator for 2 h or until cells had metabolized the substrate to MTT formazan (blue crystals). The medium was removed, 50 μL DMSO added to each well and the absorbance photometrically measured at 570 nm using the Tecan Sunrise Remote plate reader. Each condition was measured in triplicates. Cells treated with 70% ethanol served as background control.

For LDH assay, the lactate dehydrogenase release was determined 72 h p.i. by transferring 50 µL of PHH cell culture supernatant per condition per well to a 96-well plate before fixation of the cells. The supernatant was mixed with an equal amount CytoTox 96 Reagent (Promega Cat. Nr. G1780) and incubated for 30 min at room temperature, before stopping with Stopping Solution and photometrically measuring the absorbance at 492 nm using the Tecan Sunrise Remote plate reader. Each condition was performed in triplicates. Untreated, uninfected cells served as positive control.

Animal experiments

*Compounds*

Yes1 kinase inhibitor was purchased from MedChemExpress (Y1KI, CH6953755, Cat. Nr. HY-135299). Y1KI was formulated as a 10 mg/ml suspension, containing 10% (v/v) DMSO, 5% (v/v) PEG-300 (Sigma), 5% (v/v) Tween-80 (Sigma) and 45% (v/v) saline; each solvent was added one by one.

*Rat HEV*

Rat HEV LA-B350 (10% liver homogenate, GenBank accession number KM516906) was kindly provided by Prof. Suzanne Emerson (formerly at the National Institute of Allergy and Infectious Diseases, National Institutes of Health, Bethesda, MD, USA).

In vivo *rat experiments*

Female athymic nude rats (Hsd:RH-Foxn1rnu rats, Rattus norvegicus) were purchased from Envigo and kept per two in individually ventilated isolator cages (IsoCage N Bio-containment System, Tecniplast) at 21 °C, 55% humidity and 12:12 day/night cycles. Housing conditions and experimental procedures were approved by the ethics committee of animal experimentation of KU Leuven (license P003/2021). During the animal experiments, rats were weighted and checked for clinical signs every day until the end of the experiment. At the end of the animal experiments, rats were humanely killed by intraperitoneal (i.p.) injection of pentobarbital (Dolethal, Vetoquinol).

The HEV infection model of rat HEV has been described before ^2,4^. In short, two groups (n=7 for vehicle and n=5 for Y1KI) of athymic nude rats of 3–7 weeks old were infected by intravenous injections of a 1% liver homogenate of rat HEV LA-B350 (200 µL; containing approximately 1×10^6^ viral RNA copies) in the tail vein on day 0 post-infection (0 d p.i.). Rats were treated by oral gavage with either the vehicle or Y1KI at or 50 mg/kg/dose once daily (QD). Treatment started two hours before infection and continued until 12 d p.i.. Rats were weighed and monitored daily for the appearance of disease signs. From 0‒12 d p.i., feces were collected every 4 days (0, 4, 8 and 12 d p.i.). After euthanasia at 12 d p.i., liver, intestine, spleen and blood were collected and viral RNA was isolated and quantified by RT-qPCR as described previously ^2,4^.

Statistical Analysis and Software

Statistical analysis was done by using GraphPad Prism version 9.12 for Windows (www.graphpad.com). Fifty percent inhibitory concentrations were calculated employing a four parameter log logistic non-linear regression model in GraphPad Prism. To test significance of mean differences, either student t-test, one-way ANOVA followed by Dunnett’s multiple comparison test or two-way ANOVA followed by Šídák’s multiple comparison test were used, p values < 0.05 (*), <0.01 (**), <0.001 (***) and <0.0001 (****) were considered statistically significant. p values >0.05 were considered non-significant (ns). For image analysis Fiji-ImageJ version 1.54f ^5^ (www.imagej.net/ij/) and CellProfiler version 4.2.1 (www.cellprofiler.org) were used. For calculating synergy scores and plots, R version 4.2.1 was used with the package Synergyfinder (version 3.8.2)^6^. Further graphics were prepared using GraphPad Prism (version 9.12) for Windows (La Jolla, CA, USA, www.graphpad.com) and Adobe Illustrator (version 26.0.3) (www.adobe.com).

**Supplementary Figures and Tables**


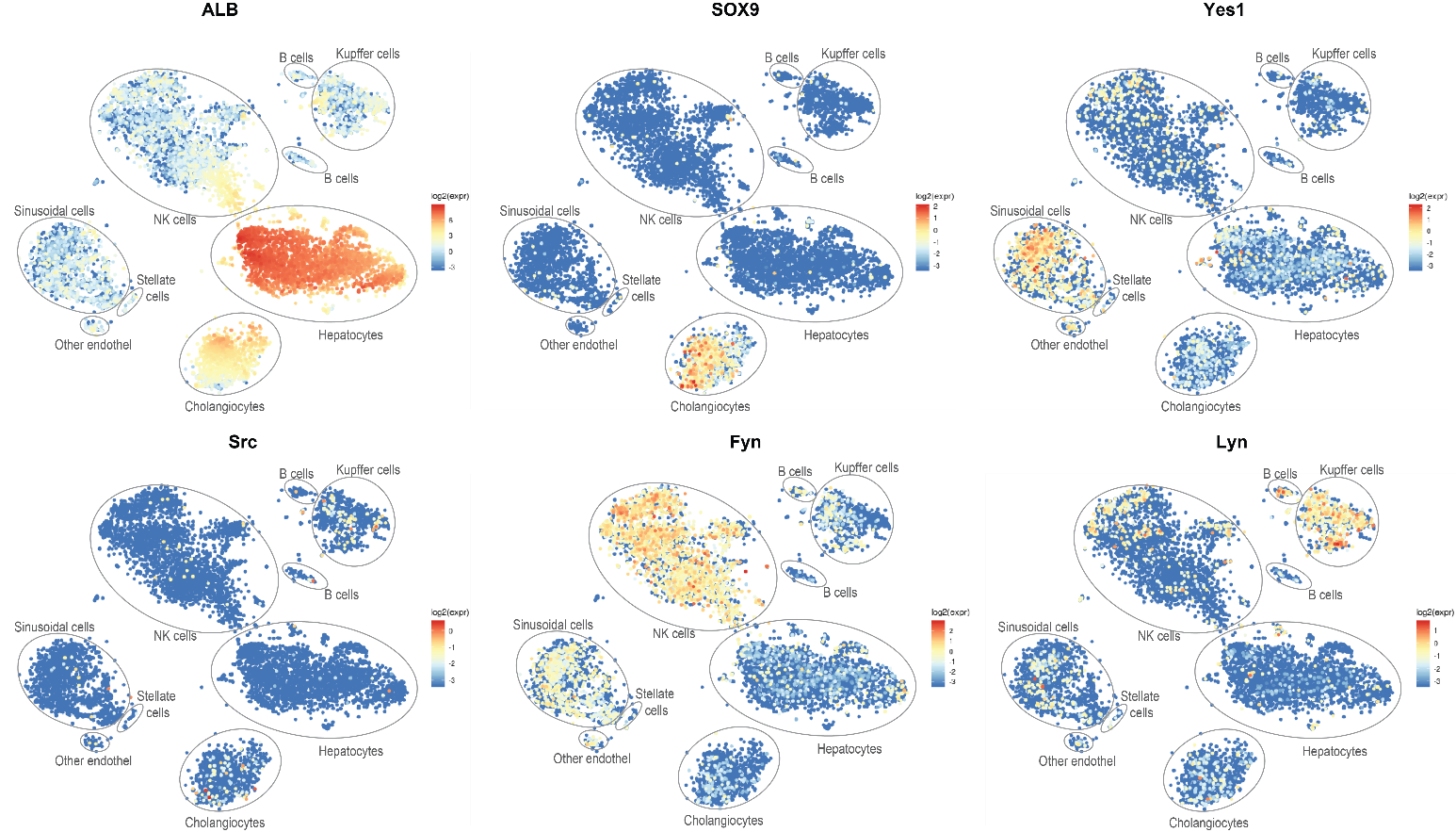
***Supplemental Figure 1:*** *Yes1 is expressed endogenously in the human liver. T-distributed stochastic neighbor-embedding (t-SNE) plots highlighting mRNA expression of ALB, SOX9, Yes1, Src, Fyn, and Lyn across all cells of healthy human liver tissue. The color represents the gene expression according to the corresponding legend as log2 value of the expression. Cell type annotation was transferred from Aizarani et al.^7^*


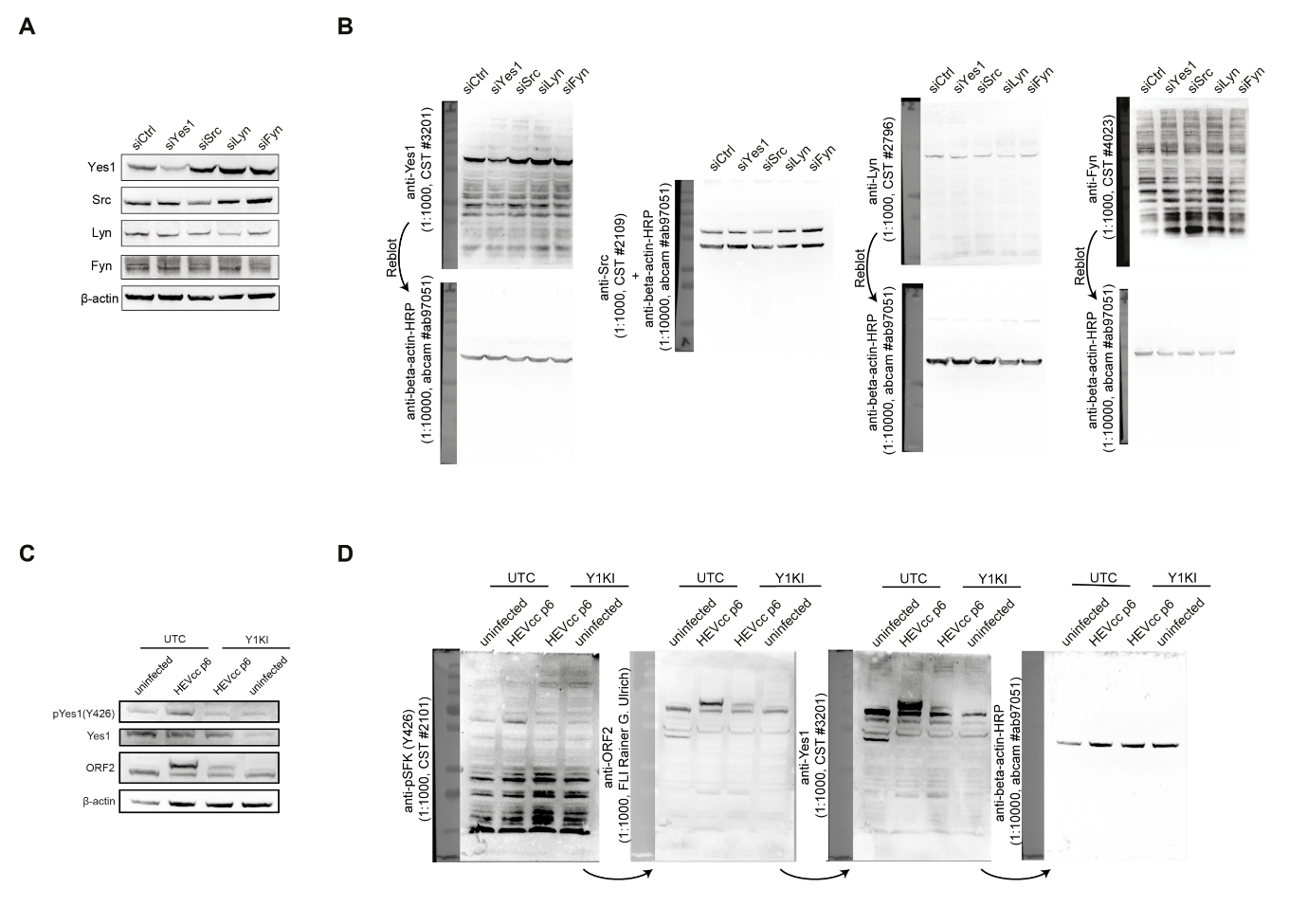


***Supplementary Figure 2:*** *Original images of all western blots.* ***(A)*** *Yes1, Src, Lyn, Fyn and β-actin expression in HepG2/C3A cells two days post transfection with siRNA targeting Yes1, Src, Lyn, Fyn or siCtrl, respectively, analyzed via western blot.* ***(B)****Original western blots of (A).* ***(C)****Expression of Yes1 and phosphorylation status at Y426 in HepG2/C3A cell lysates uninfected or infected with HEVcc p6 non-env. 3 d p.i. of untreated control cells (UTC) or treated with 1 µM Y1KI, respectively, corresponding to figure 2C.* ***(D)****Original western blots of (C). Left lanes of each blot: Marker: PageRuler Plus Prestained Protein Ladder. Reblots were performed as indicated using a mild ReBlot buffer.*


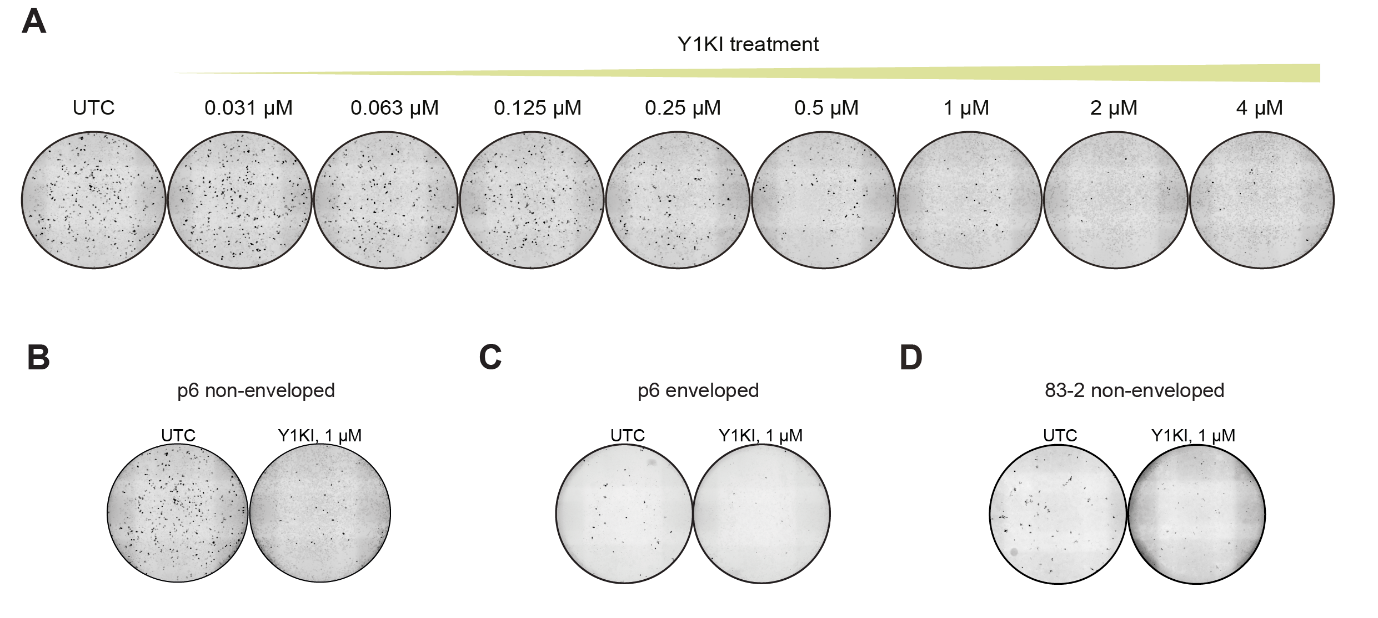


***Supplemental Figure 3: (A)*** *Full well images of HEV infections (visible in black) of HepG2/C3A cells infected with HEVcc p6 non-env. under simultaneous treatment of the indicated concentration of Y1KI corresponding to quantification in figure 2D. Cells were fixed and stained for ORF2 protein at three d p.i. ORF2+ cells are indicated in black.* ***(B‒D)*** *Full well images of HEV infections of HepG2/C3A cells infected with HEVcc p6 non-env. (B), HEVcc p6 env. (C) or HEVcc 83-2 non-env. (D) under simultaneous treatment 1 µM Y1KI. Images were stitched and processed using Fiji.*

***
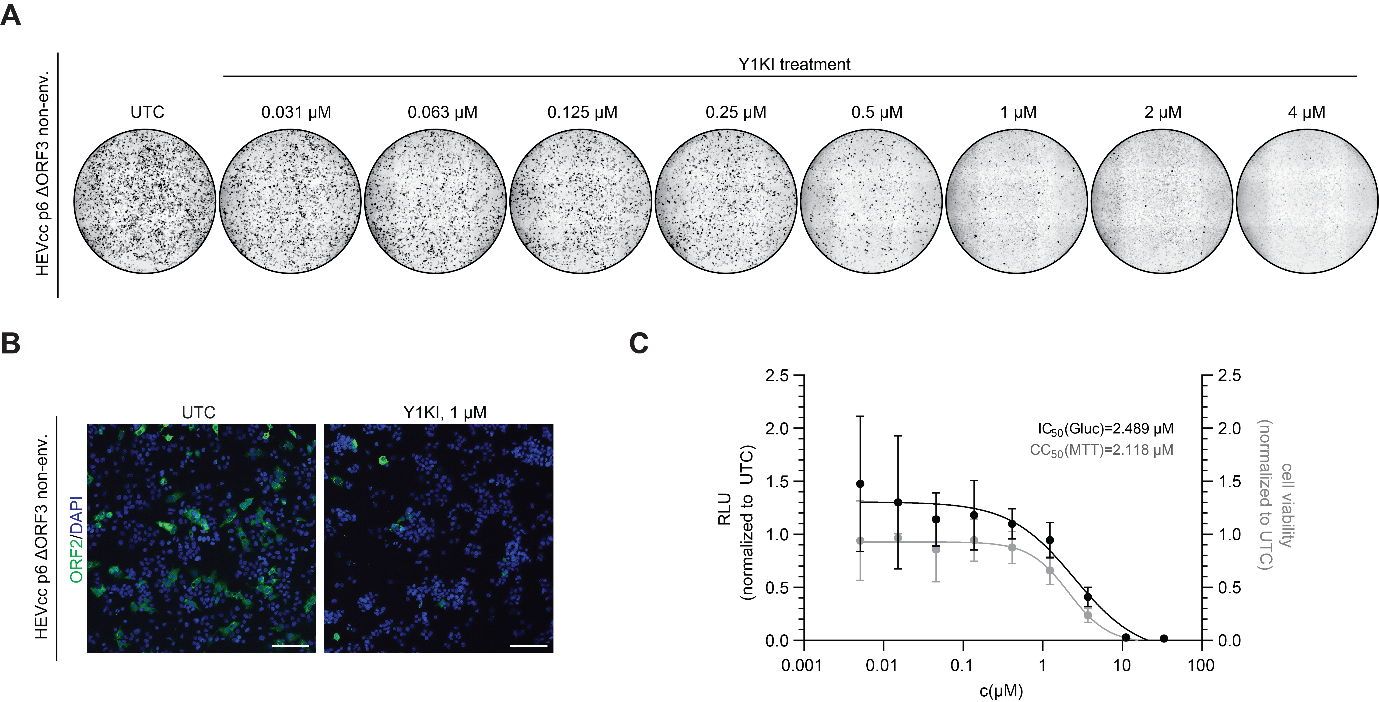
Supplemental Figure 4: (A)****Full well images of HEV infections (black) of HepG2/C3A cells infected with HEVcc p6 ΔORF3 non-env. under simultaneous treatment of the indicated concentration of Y1KI Cells were infected two days post transfection and stained for ORF2 protein at three d p.i. Images were stitched and processed using Fiji. ORF2+ cells are indicated in black.* ***(B)****Representative fluorescence image of HepG2/C3A cells infected with HEVcc p6 ΔORF3 non-env. of UTC or under simultaneous treatment of 1 µM Y1KI Scale bars = 100 µm.* ***(C)****HEV replication levels (black) at 72 h p.e. under simultaneous treatment of the indicated concentrations of Y1KI measured using the HEV p6 subgenomic replicon system in HepG2/C3A. Cell viability (grey) measured using an MTT assay. For all graphs, mean and SEM from at least three independent experiments, each performed in triplicates are depicted. Dose-dependent treatment and 50% percent inhibitory concentrations (IC_50_ and CC_50_) were calculated employing a four parameter log logistic non-linear regression model (C).*


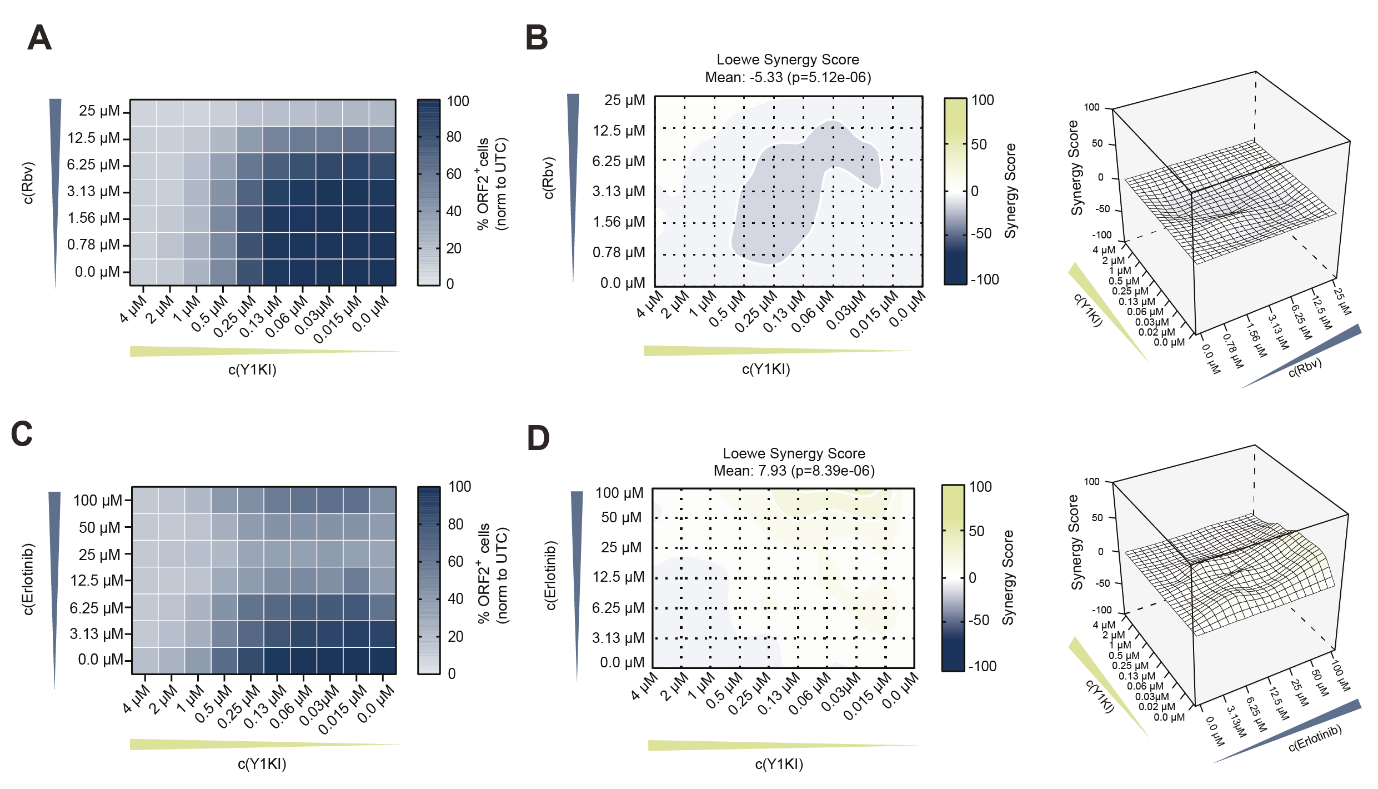


***Supplemental Figure 5: (A)*** *Combination treatment of the indicated concentrations of Ribavirin and Y1KI simultaneously to infection with HEVcc p6 non-env in HepG2/C3A cells. Quantification of HEV infection levels after staining for ORF2 protein at 3 d p.i.* ***(B)*** *Loewe Synergy Scores based on (A) plotted as 2D map (left) and 3D surface graph (right).* ***(C)*** *Combination treatment of the indicated concentrations of Erlotinib and Y1KI simultaneously to infection with HEVcc p6 non-env in HepG2/C3A cells. Quantification of HEV infection levels after staining for ORF2 protein at 3 d p.i.* ***(D)*** *Loewe Synergy Scores based on (C) plotted as 2D map (left) and 3D surface graph (right). For all graphs, mean values from three independent experiments are depicted, each performed in triplicates. Synergy Scores were calculated using the Synergyfinder package in R.*


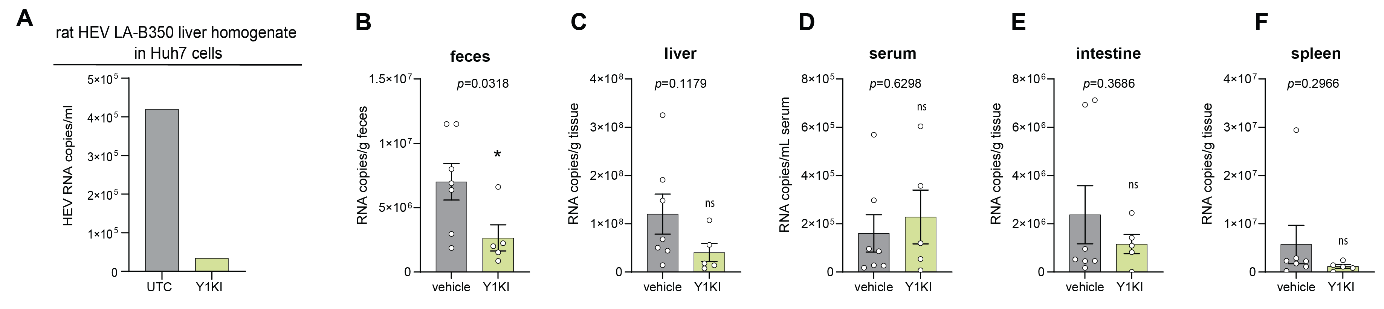


***Supplemental Figure 6: (A)****Quantification of ratHEV RNA copy numbers via RT-qPCR at 10 d p.i. in cell culture supernatant in rat HEV LA-B350 liver homogenate infected Huh7 cells. Depicted is mean of one biological replicate measured in duplicates.* ***(B–F)*** *Rat HEV RNA copy numbers normalized to 1 g tissue/feces or 1 mL serum from all rats of the respective group from 12 d p.i.* *Mean values ±SEM of all rats tested are depicted. To test significance of mean differences, student t test was used. p-values <0.05 (*), p-values >0.05 were considered to be not significant (ns).*

*Supplementary Table 1: Details of the primary human hepatocyte donors obtained from Primacyt.*

| **Lot#** | **Donor Gender** | **Donor Age** | **Race** | **Pathology** | **Cells Seeded on 24-well plate** |
| --- | --- | --- | --- | --- | --- |
| CHM2221 | Male | 51 | Caucasian | Cholangiocarcinoma | 4.31×10^5^ |
| CHM2225 | Male | 73 | Caucasian | Hepatocellular Carcinoma | 3.82×10^5^ |
| NHM2251 | Male | 47 | Hispanic | - | 3.37×10^5^ |
| BHum15052 | Male | 54 | Caucasian | Colorectal cancer with hepatic metastases | 4.17×10^5^ |

*Supplementary Table 2: List of used siRNAs (Ambion/ Thermofisher).*

| Target | Entrez Gene ID | ID | Validated? | Target | Entrez Gene ID | Cat. Nr. | Validated? |
| --- | --- | --- | --- | --- | --- | --- | --- |
| ABL1 | 25 | s865 s864 | Yes | MAPK3 | 5595 | s11140 s11141 | Yes |
| BMPR2 | 659 | s2045 s2046 | Yes | MAPK7 | 5598 | s11149 s11151 | Yes/No |
| CAMK4 | 814 | s2355 s2357 | Yes | MOK | 5891 | s19  s18 | No |
| CDC7 | 8317 | s15828 s15826 | Yes | PTK2 | 5747 | s11485 s11484 | Yes/No |
| CDC42 | 998 | s227090 s2766 | No | RB1 | 5925 | s522  s523 | Yes |
| CDK6 | 1021 | s53  s51 | Yes | ROCK1 | 6093 | s12097 s12098 | Yes |
| CHEK1 | 1111 | s504  s503 | Yes | RPS6KA | 6195 | s12274 s12275 | Yes |
| CHEK2 | 11200 | s533979 s533980 | No | SIK3 | 23387 | s225956 s225958 | No |
| EGFR | 1956 | s564 s565 | Yes | TNK2 | 10188 | s19850 s19852 | No |
| ESYT1 | 23344 | s23607 s23606 | No | TSG101 | 7251 | s14439 s14440 | No |
| FGFR1 | 2260 | s5165 s5164 | Yes | Yes1 | 7525 | s14957 s14956 | Yes |
| FGFR2 | 2263 | s5173 s5175 | No | ZAP70 | 7535 | s533545 s14974 | No |
| FLT3 | 2322 | s5290 s5291 | No | Src | 6714 | s13413 s13414 | No/Yes |
| ICK | 22858 | s22514 s22516 | Yes | Lyn | 4067 | s8355 s8356 | Yes |
| MAP2K6 | 5608 | s11181 s11180 | Yes | Fyn | 2534 | s5434 s5435 | Yes |
| MAP2K7 | 5609 | s11184 s11182 | No |  |  |  |  |

**References**

1. Fu R, Engels Z, Weihs JA, et al. A high-content RNA-based imaging assay reveals integrin beta 1 as a cofactor for cell entry of non-enveloped hepatitis E virus. *bioRxiv*. 2023;doi:10.1101/2023.10.27.564362

2. Zhang X, Cremers N, Hendrickx S, et al. Establishment of a robust rat hepatitis E virus fecal-oral infection model and validation for antiviral studies. *Antiviral Res*. Aug 2023;216:105670. doi:10.1016/j.antiviral.2023.105670

3. Shukla P, Nguyen HT, Faulk K, et al. Adaptation of a genotype 3 hepatitis E virus to efficient growth in cell culture depends on an inserted human gene segment acquired by recombination. *J Virol*. May 2012;86(10):5697-707. doi:10.1128/JVI.00146-12

4. Debing Y, Mishra N, Verbeken E, Ramaekers K, Dallmeier K, Neyts J. A rat model for hepatitis E virus. *Dis Model Mech*. Oct 1 2016;9(10):1203-1210. doi:10.1242/dmm.024406

5. Schindelin J, Arganda-Carreras I, Frise E, et al. Fiji: an open-source platform for biological-image analysis. *Nat Methods*. Jun 28 2012;9(7):676-82. doi:10.1038/nmeth.2019

6. Zheng S, Wang W, Aldahdooh J, et al. SynergyFinder Plus: Toward Better Interpretation and Annotation of Drug Combination Screening Datasets. *Genomics Proteomics Bioinformatics*. Jun 2022;20(3):587-596. doi:10.1016/j.gpb.2022.01.004

7. Aizarani N, Saviano A, Sagar, et al. A human liver cell atlas reveals heterogeneity and epithelial progenitors. *Nature*. Aug 2019;572(7768):199-204. doi:10.1038/s41586-019-1373-2
